# Experimental and *In Silico* Modelling Analyses of the Gene Expression Pathway for Recombinant Antibody and By-Product Production in NS0 Cell Lines

**DOI:** 10.1371/journal.pone.0047422

**Published:** 2012-10-10

**Authors:** Emma J. Mead, Lesley M. Chiverton, Sarah K. Spurgeon, Elaine B. Martin, Gary A. Montague, C. Mark Smales, Tobias von der Haar

**Affiliations:** 1 School of Biosciences, University of Kent, Canterbury, United Kingdom; 2 School of Engineering and Digital Arts, University of Kent, Canterbury, United Kingdom; 3 Centre for Molecular Processing, University of Kent, Canterbury, United Kingdom; 4 School of Chemical Engineering and Advanced Materials, Newcastle University, Newcastle, United Kingdom; Simon Fraser University, Canada

## Abstract

Monoclonal antibodies are commercially important, high value biotherapeutic drugs used in the treatment of a variety of diseases. These complex molecules consist of two heavy chain and two light chain polypeptides covalently linked by disulphide bonds. They are usually expressed as recombinant proteins from cultured mammalian cells, which are capable of correctly modifying, folding and assembling the polypeptide chains into the native quaternary structure. Such recombinant cell lines often vary in the amounts of product produced and in the heterogeneity of the secreted products. The biological mechanisms of this variation are not fully defined. Here we have utilised experimental and modelling strategies to characterise and define the biology underpinning product heterogeneity in cell lines exhibiting varying antibody expression levels, and then experimentally validated these models. In undertaking these studies we applied and validated biochemical (rate-constant based) and engineering (nonlinear) models of antibody expression to experimental data from four NS0 cell lines with different IgG4 secretion rates. The models predict that export of the full antibody and its fragments are intrinsically linked, and cannot therefore be manipulated individually at the level of the secretory machinery. Instead, the models highlight strategies for the manipulation at the precursor species level to increase recombinant protein yields in both high and low producing cell lines. The models also highlight cell line specific limitations in the antibody expression pathway.

## Introduction

Mammalian cell lines have been used industrially for several decades for the production of complex, high value recombinant therapeutic proteins. They are preferred over other expression systems largely because of their ability to correctly fold, assemble and undertake the required post-translational modifications that decorate recombinant proteins of eukaryotic origin [1], [2]. Biotherapeutics produced in mammalian expression systems include recombinant monoclonal antibodies (mAbs) [2] and plasma proteins [1]. As the demand for such protein based therapies has increased, so have the yields obtained from mammalian expression systems, with current product yields more than a 100-fold greater than those achieved 20–30 years ago [2], [3], [4]. Most of this increase in yield has come through improvements in culture media composition and feeding regimes [2], and/or via improved screening strategies to identify cell lines that obtain and maintain higher biomass [5].

An alternative to improving biomass yield or viable cell concentration is to enhance the cell specific productivity (or amount of product produced per cell per unit time, qP). Approaches to improve qP include direct cell engineering (see below), culture additives (e.g. sodium butyrate [6]), or manipulation of the culture environment (e.g. change in culture temperature [7], [8]). The cellular mechanisms by which such approaches improve qP are poorly understood.

There have been various approaches investigated to improve the cell specific productivity of mammalian cell lines by direct manipulation of the cellular machinery itself, for example by over-expression or knockdown of specific targets [9]. Particular targets investigated to date with a view to improving qP in mammalian cell lines include anti-apoptotic genes [10], [11], [12], [13], cell cycle related genes [14], [15], [16], the folding and assembly machinery in the endoplasmic reticulum [17], [18], [19], [20], [21], [22], and the translational [23], [24], [25] and secretory machinery [26]. However, such approaches to improving qP in mammalian cell lines have largely resulted in conflicting or disappointing results. While these attempts at manipulating the cellular machinery are based upon our knowledge of the general requirements for, and bottlenecks in, protein synthesis and secretion in mammalian cells, we do not currently have a complete understanding of the recombinant gene expression pathway and the intricate interactions between the various cellular processes that are required to work in symphony to give and define a highly productive recombinant cell line.

In the specific case of monoclonal antibodies produced from mammalian cells, a number of groups have attempted to define the limitations upon their cell specific production (qmAb), and hence identify rational targets for cell engineering, using ‘omic’ profiling of cell lines exhibiting differing qmAbs [27], [28], [29], [30], [31], [32], [33], [34], [35], [36]. These studies have largely focussed on either transcriptomic or proteomic profiling, and generally show that there are many cell line specific differences in gene expression activity that correlate with qmAb. Moreover, there are specific classes or families of proteins that also correlate with qmAb in their expression levels. A problem with interpreting these studies is the difficulty in deciding whether observed changes in gene expression are the result of high qmAb, underpin high qmAb, or are a non-specific consequence of the various cellular processes that show changes in gene expression correlating with qmAb. As such, whilst these studies have furthered our understanding of cellular processes that underpin high qmAb, they have generally not been able to clearly define these processes, nor to quantify their individual contribution to antibody expression.

Another approach to identifying cellular constraints upon qmAb by which engineering strategies could be devised and validated is to use model-based approaches [37]. Such approaches can consider the whole gene expression pathway and the contribution of the different cellular processes to it, allowing both the identification of cellular bottlenecks, and the prediction of how engineering strategies or manipulation of specific steps might impact upon qmAb. A small number of such studies have now shown the proof of concept of such an approach [4], [38], [39]. Indeed, we have previously used a luciferase model system to show that the quantitative determination of gene expression intermediates can be a powerful tool for analysing cell-line specific limitations in gene expression pathways [40]. Here we set out to apply the same strategy to analysing the expression of a model IgG4 antibody. Antibodies are the fastest growing group of the high-value biopharmaceuticals [41], and have a complex gene expression pathway that involves the synthesis of two heavy and two light chain polypeptides, followed by folding of the polypeptide chains, assembly into the tetrameric antibody structure, and secretion of the final product.

There are four subclasses of the IgG isotype (IgG_1–4_) and the folding and assembly of the IgG1 and IgG2 isotypes have been described in some detail (e.g. see [42]. However, the folding pathway(s) of the IgG4 isotype is currently not well characterised. Moreover, antibody secretion is usually accompanied by the secretion of contaminant folding intermediates and off-pathway by-products, and we wished to understand the relationship between the productive and non-productive pathways in more detail. Here we describe the measurement of key parameters throughout the IgG4 gene expression pathway in NS0 cells of varying productivity, and then use this information and our knowledge of the synthesis, folding and assembly pathways of IgGs to develop models that describe the gene expression pathway and the limitations upon this in four different NS0 cell lines. We subsequently validated these models by successfully predicting qmAb for a naive cell line. Our models highlight the cell line specific nature of limitations on qmAb but also highlight potential engineering approaches to remove cellular limitations upon gene expression.

## Results

**Figure 1:**
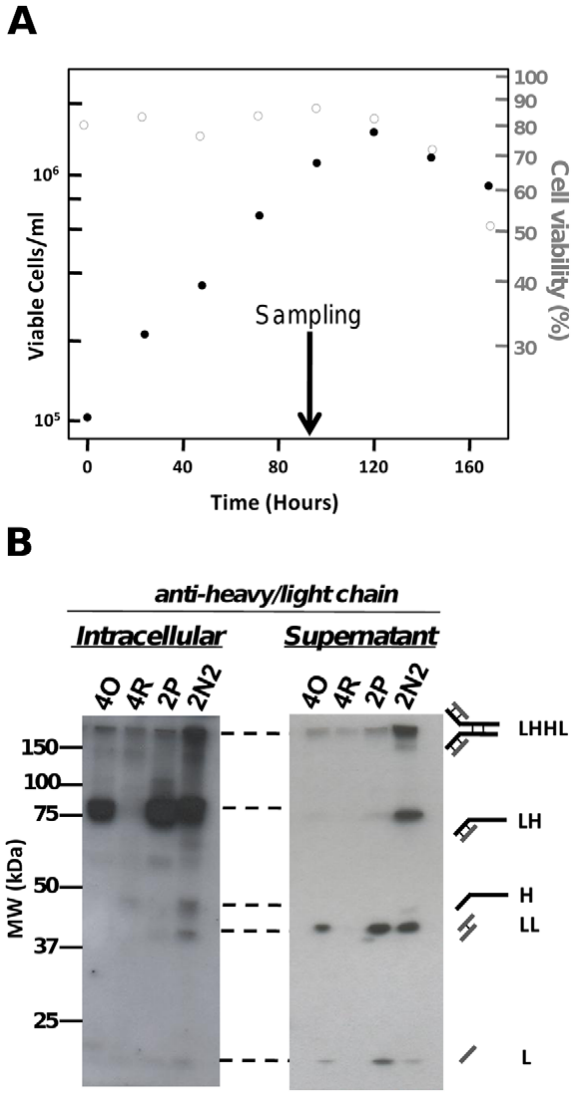
Antibody-related species detected by western analysis in the different cell lines. **A.** Example growth curve of cell line 2N2. A sampling point was chosen below the maximum viable cell concentration, where cell-specific productivity was observed to be stable. (Full growth curves are available in File S1) **B.** The different species that could be observed in intracellular or supernatant samples were identified by western blotting with a polyclonal antibody able to detect both heavy chain and light chain species. The species are (from top) assembled antibody (LHHL), half antibody (LH), single heavy chain (H), light chain dimer (LL) and single light chain (L). For clarity of species observation intracellular and supernatant blots are representative and not of the same exposure time, quantitative data was obtained by western blotting alongside internal standards and is given in Table 1. Full data tables are provided in File S5.

We investigated four recombinant GS-NS0 cell lines which are engineered to express a chimeric cB72.3 IgG4 molecule and which had previously been shown to exhibit different qmAbs [35]. These cell lines were grown under batch culture conditions as described in the methods section. We initiated the study by analysing which polypeptide and protein intermediates of the antibody folding pathway were observable in our cell lines using a western blotting approach. For sampling we chose a time point 96 hours after seeding of the cells during mid-exponential growth phase (Fig. 1A). Up to this point in culture, growth rate, cell viability and productivity of each of the four cell lines investigated was constant and these parameters did not start to decline until approximately 120 h of batch culture. The combination of constant growth rate and constant productivity at the sampling point suggests that the cells are internally in a steady state (File S1). This is consistent with many published modelling studies relying on the same assumption [4], [10], [11], [12], [38], [43].

**Table 1:** Summary of experimentally determined parameters.

		2N2	2P	4O	4R	
Steady state levels	H.RNA	781	250	71	137	molecules*cell^−1^
	L.RNA	60611	41165	741	550	
	H	4.7×10^6^	2.5×10^6^	<0.1×10^6^	2.3×10^6^	
	L	1.7×10^6^	1.4×10^6^	1.1×10^6^	1.4×10^6^	
	LL	1.2×10^6^	0.8×10^6^	0.7×10^6^	0.5×10^6^	
	HL	160×10^6^	60×10^6^	10×10^6^	>0.1×10^6^	
	HH	<0.1×10^6^	<0.1×10^6^	<0.1×10^6^	<0.1×10^6^	
	LHH	<0.1×10^6^	<0.1×10^6^	<0.1×10^6^	<0.1×10^6^	
	LHHL	12×10^6^	2.6×10^6^	1.4×10^6^	2.3×10^6^	
secretion rates	qL	37×10^6^	1.6×10^6^	0.4×10^6^	<0.1×10^6^	molecules *cell^−1^*h^−1^
	qLL	12×10^6^	1.0×10^6^	0.1×10^6^	<0.1×10^6^	
	qmAb	2.9×10^6^	0.4×10^6^	0.1×10^6^	<0.1×10^6^	
	qHL	11×10^6^	0.7×10^6^	0.1×10^6^	<0.1×10^6^	
rate constants	Growth	0.020	0.025	0.036	0.025	h^−1^
	H.RNA decay	<0.025	0. 400	0.038	0.043	
	L.RNA decay	<0.025	0.109	0.026	0.026	

**H  = ** heavy chain; **L** =  light chain.

When analysing intracellular material from the four cell lines by western analysis (with qmAbs (molecules*cell^−1^*h^−1^) as follows: 2N2–2.6×10^6^, 2P –0.4×10^6^, 4O –0.1×10^6^ and 4R – <0.1×10^6^) we could distinguish a number of bands which reacted with a polyclonal anti-human IgG whole antibody, consisting of a mixture of heavy and light chain species specific antibodies (Fig. 1B). In order to maintain model fidelity only bands which could be clearly assigned to a given antibody species were used in model construction. A fully assembled antibody consists of two heavy and two light chains held together by disulphide bridges, and probing with the individual heavy- and light-chain antibodies allowed us to unambiguously identify 2 major and 3 minor bands (Fig. 1B), in agreement with other western analysis of the IgG4 cB72.3 molecule [30], [44]. These bands correspond to full antibody (LHHL where L  =  light chain and H  =  heavy chain), half-antibody (HL), free heavy- (H) and free light- (L) chain, and light-chain dimer (LL). In the cell-culture supernatant western blotting using the polyclonal anti-human IgG whole antibody identified four species that corresponded to full and half antibody, free light chain, and light chain dimer (Fig. 1B and File S2). It was obvious even from this simple analysis that there were differences in the abundance of the species present between the cell lines.

**Figure 2:**
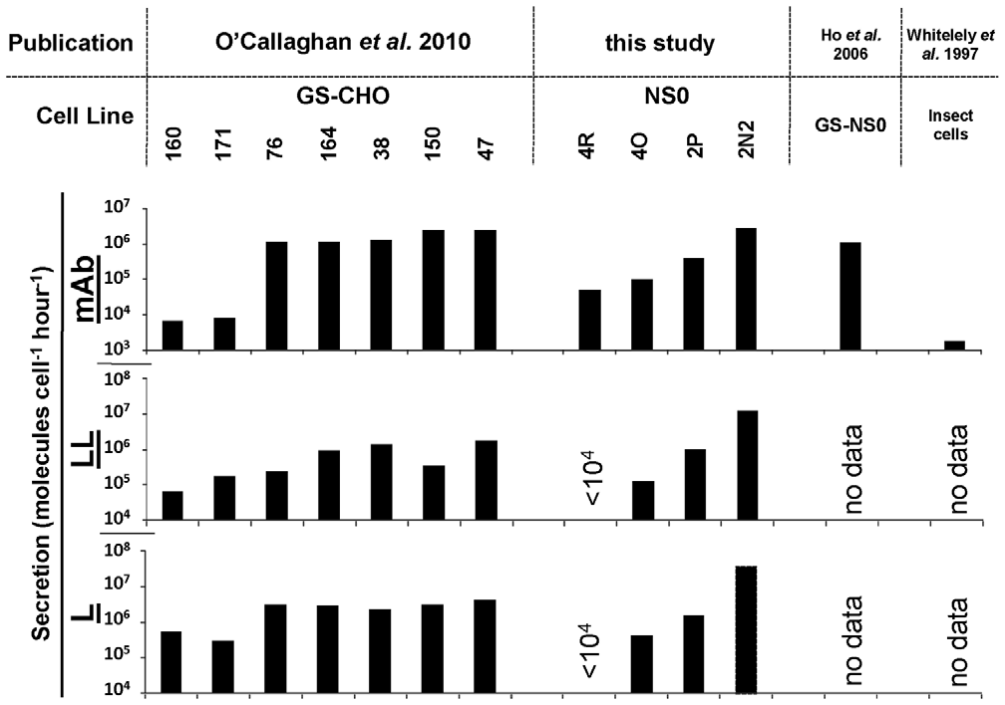
Comparison of productivity in the current model system with other reported expression systems. Secretion rates observed in the cell lines used in this study are compared to a number of published rates for various eukaryotic expression systems [4], [38], [43]. L (light chain), LL (light chain dimer), mAb (assembled monoclonal antibody).

The data shown in Fig. 1B indicate the relative abundance of the species observed at the sampling time point. Additional monitoring of the change in abundance of the different supernatant species over time allowed us to assign secretion rates for each of these, and to compare secretory activity in our cell lines to previously published data ([4], [38], [43], Fig. 2). We conclude from this analysis that both in terms of secretion of the fully formed monoclonal antibody, and of the two major by-products (free light chain L and light-chain dimer LL), the secretion parameters lie within the range reported by others for IgG secretion from mammalian and insect cells and hence represent an appropriate model set. Further, this analysis confirmed that the four cell lines cover a range of qmAb values (Fig. 2) that would facilitate our modelling studies.

**Figure 3:**
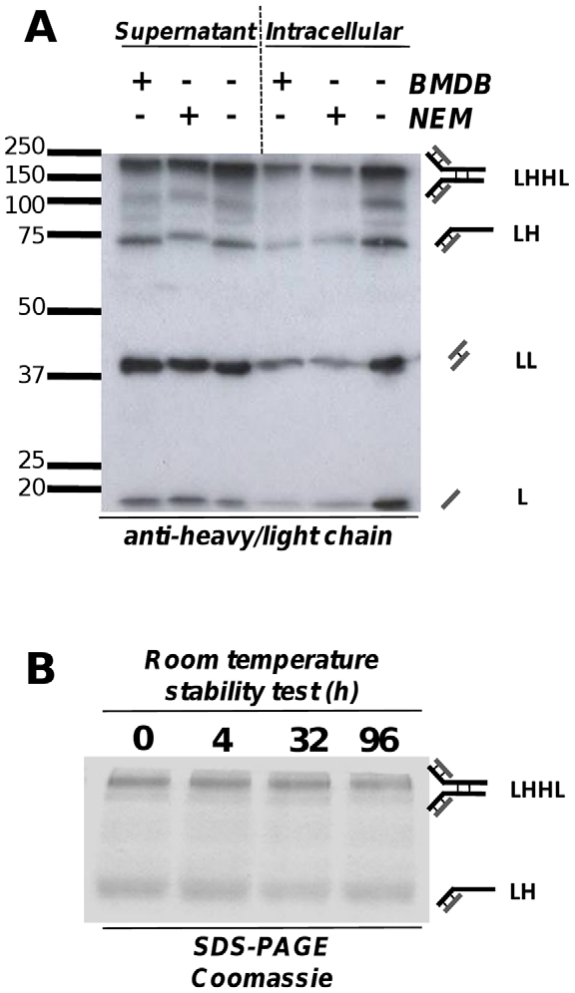
Origin of the HL species. **A.** Treatment of 2N2 samples with oxidising agents does not alter the ratio of half-antibody to full mAb. BMDB (1,4-bismaleimidyl-2,3-dihydroxybutane), NEM (N-ethylmaleimide), LHHL (assembled antibody), LH (half antibody species), LL (light chain dimer), L (light Chain). **B.** In cell culture supernatants the ratio of half- to full antibody does not change upon prolonged incubation at room temperature. Both analyses indicate that the half antibody species does not result from decay of the full antibody.

In the supernatant we also observed a species which we identified as half-antibody based on its apparent molecular weight and its reactivity with anti-heavy and anti-light chain antibodies (File S3). Intracellularly this species is a potential antibody assembly intermediate, however in the supernatant it is considered a by product [45]. O'Callaghan *et al*. also observed this species in CHO cells, and initially ascribed this to the reductive decay of full antibody into two half-molecules [4]. In a more recent study they suggest this is generated as a result of a slow rate of redox recycling of half antibody into full antibody within the ER [46]. However, in contrast to the situation in CHO cells, we did not observe any change in the half-antibody: full-antibody ratio if our samples were treated with different reducing agents that would prevent Fab arm exchange (Fig. 3A). Moreover, this ratio did not change upon prolonged incubation of purified antibody at room temperature (Fig. 3B), making it unlikely that in our case this species arose as the result of a decay process. On the other hand, the abundance of this species in the supernatant was dependent on the general cellular export activity (see analyses below), and we therefore conclude that in our NS0 cell lines, half-antibody is exported from the cell in a similar manner to the other three supernatant species.

IgG4 antibodies undergo Fab arm exchange reactions [45], [47] and we note that although this species in the supernatant appears as a half-antibody of 75 kDa molecular weight on denaturing SDS-PAGE gels, it may not be exported in this form. Gel filtration experiments conducted on isolated antibodies show that under non-denaturing conditions this species elutes with an apparent molecular weight of 150 kDa (data not shown) [44]. This indicates that in solution the IgG4 half-molecule (HL) actually exists as non-covalently bound full antibody and it is likely that this species is exported in this form. Indeed, the inter-heavy chain disulfide bonds of IgG4 have been reported to be in equilibrium with intra-chain disulfide bonds resulting in some of the IgG4 having non-covalently linked heavy chains [48]. The levels of this species were reported to be expression system dependent and this may explain why much lower levels of half antibody for the same IgG4 were reported when expressed in CHO cells as in the study of O'Callaghan [4] compared to our observations in NS0 cells.

Following the basic characterisation of observable species in the supernatant, we determined absolute levels of observable intracellular protein intermediates by quantitative western blotting and comparison against a standard of purified full-length antibody and antibody-fragments of known concentrations (Table 1 and File S2). Such an approach has previously been demonstrated to be accurate for quantitatively determining protein levels [49]. Absolute amounts of heavy and light chain mRNA were also determined using qRT-PCR using *in vitro*-transcribed mRNA as a quantitative reference (Table 1). The steady state levels showed a large excess of light chain mRNA compared to heavy chain mRNA in all cell lines, but this was not reflected at the polypeptide level where the amount of intracellular heavy chain, and of light chain and light chain dimer (LL) combined, were of a similar magnitude (Table 1).

In our previous analyses [40], we combined steady-state abundance data such as those determined here with information on turnover rate constants to form the basis for a comprehensive characterisation of a simple gene expression pathway. In the case of monoclonal antibodies, the determination of turnover rate constants is difficult because, upon inhibition of translation, the existing species are processed by a number of pathways including protein turnover, formation of higher-order complexes, and export. Consequently, we observed that when we inhibited translation by addition of cycloheximide, the abundance of individual species changed with complex rates that could not be fitted to simple first- or second-order rate constants (data not shown). The information on our antibody-producing cell-lines is thus limited to steady-state abundance of intracellular species and to secretion rates. Given this limited information, we decided to investigate how far *in silico* modelling approaches could assist the characterisation of antibody expression pathways in the different cell lines.

**Figure 4:**
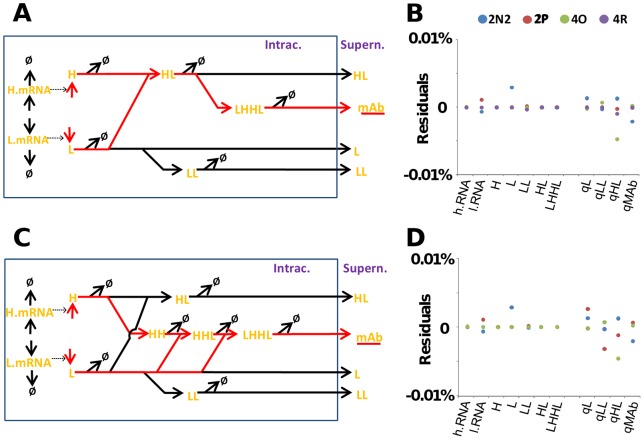
The fit of our models to the observed steady-state values determined experimentally. Different antibody assembly models were fitted to the different observed steady-state data given in Table 1. Panels on the left-hand side (A and C) show the assembly routes for the two different models, with productive reactions (those leading to the formation of full antibody) displayed in red. The panels on the right-hand side (B and D) indicate the difference between the best-fitting parameter set in the biochemical models and the observed parameter values. The two models, as well as a model combining the two assembly pathways, can all reproduce the experimentally observed data for all four cell-lines with high similarity. Similar results were obtained for the engineering models. H.mRNA (heavy chain mRNA), L.mRNA (light chain mRNA), LHHL (assembled antibody), LH (half antibody species), H (heavy chain), HH (heavy chain dimer), HHL (heavy chain dimer +1 light chain), LL (light chain dimer), L (light Chain), mAb (assembled secreted antibody), qmAb (mAb secretion rate), qHL (HL secretion rate), qLL (LL secretion rate), qL (L secretion rate).

In defining our mathematical models we considered reports that IgGs can assemble *via* two principal routes, either by formation of a half-antibody intermediate (HL) from which the full antibody is assembled via the association of two HL molecules (“HL” pathway where H  =  heavy chain polypeptide and L  =  light chain polypeptide, H->HL->(HL)_2_), or by the initial formation of a heavy-chain dimer to which then sequentially recruits two light chains (“HH” pathway, H->H_2_->H_2_L->H_2_L_2_) [42], [50]. The assembly pathways for several murine IgGs have been elucidated and predominantly proceed through the HH pathway for IgG_1_ and IgG_2a_, and the HL pathway for IgG_2b_ and IgM [42]. Sears and colleagues also suggested that whilst these might be the major pathways for assembly, it was possible that assembly occurred via concurrent pathways [50]. Despite such studies, there is no definitive work yet published on the assembly routes of chimeric antibodies (such as the cB72.3 antibody in this study), human antibodies, or IgG4 antibodies in general. Our observations of species levels did not allow us to unambiguously define which assembly pathway was utilised in our cell lines and we therefore modelled the two potential assembly pathways independently (Fig. 4) alongside a combined model.

The two models we used contain 11 (“HL”) and 13 (“HH”) abundance parameters, respectively, as well as 19 (“HL”) and 23 (“HH”) rate parameters (Fig. 4 and File S3). We were able to experimentally determine all but two of the abundance parameters, and placed upper limits on the two undetectable species (HH and HHL in the “HH” model). In contrast, we only determined five rate parameters directly (the productivity or “q” parameters for the four exported species, and the growth rate). In normal modelling terms, this limited number of determined rate parameters could mean that the models are strongly underdetermined. In order to deal with the paucity of parameters, we used two independent modelling approaches based either on systems of classic biochemical pathways, or on approaches optimised for use in the engineering field. Both models use systems of differential equations, detailed in File S3, as underlying descriptions of the model structure and are defined by the MatLab source code given in File S4.

For the biochemical rate-based model, we avoided complications resulting from the experimentally undetermined rate constants by using a single simplifying assumption, namely that intracellular protein turnover was negligible compared to the flux of molecules along the antibody assembly pathway. This assumption is likely to introduce only a small error into the model: for example, a recent study that investigated ubiquitination of the different mAb polpypeptide species in CHO cells found that only ubiquitinated full antibody was observed, that this accounted for only 0.3% of the total assembled antibody, and that the secretory machinery beyond the ER was not saturated [46]. This suggests that very little antibody or assembly intermediates are targeted for degradation and supports the use of this assumption here.

In assuming that there is zero recombinant polypeptide/protein turnover, the entire outflow of mass from the system is thereby given by the q-values of the four exported species and by the dilution through growth i.e. by the five experimentally determined rates. Following the laws of mass conservation, the molecular flux through all of the other branches of this model can then be accurately calculated. Apparent rate constants for all reactions can be calculated by dividing the steady-state fluxes through the observed steady-state levels of the respective intermediates. With the resulting parameters, this model reproduces observed steady-state values accurately.

In order to validate this approach, we also made use of alternative modelling techniques from the domain of control engineering. In the systems biology discipline it is usually assumed that mathematical models do not perfectly represent reality. Indeed, even in cases where high fidelity models are available, it is acknowledged that external factors will change parameters and external disturbances will affect the model. A key issue therefore becomes that of robustness i.e. ensuring that the system response is minimally sensitive to any perturbations in parameters of the model. In the engineering model developed to describe the NS0 cell lines, the possible interconnections between all parameters were implemented as a linear state matrix augmented with a gain matrix distributing the nonlinear interconnections within the system. Unknown rate constants for HH and HL models were determined via least squares fitting at steady-state conditions and the best-fitting parameter sets applied to our models. Residual plots of the difference between experimentally observed parameter values vs. those defined by the models show only very small differences, indicating that resulting models can reproduce experimental observations for all four cell lines robustly (Fig. 4). However, the course of the analysis established that the two models were highly ill-conditioned, with a conditioning number of 10^10^, meaning that parameter estimates have the potential to be very sensitive to small changes in the estimated rate constants. The combined model, in which HH and HL pathways are allowed to coexist, had a higher conditioning number of 10^14^, and in this case pseudo inversion was used to define the unknown parameters [51]. It was possible to determine rate constants consistent across the three models, with the results from the independent ‘HH’ and ‘HL’ sub-models being used to verify the findings for the combined model.

In terms of validating the sensitivity of the parameterisation to changes in the assumed steady-state, the performance of the model was investigated at steady-state conditions ‘close’ to those measured experimentally, where a 10% change in steady-state conditions was assumed. Other tests were also carried out at steady-state conditions far from the measured conditions (where a 100% change was assumed). The characteristics of the model behaviour were preserved and the model was found to be robust to changes in both the assumed steady-state and the parameter estimates.

For both assembly pathways, we tuned the models to reproduce our experimentally observed steady-state abundance nearly perfectly (Fig. 4). Detailed parameter values are given in Table 2. It should be noted that the rate constants emerging from our models are pseudo-first order constants which group together many detailed rate constants from the complex reactions occurring during real antibody assembly, and they therefore have limited meaning in a true biological sense. However, as we show below, they can be subsequently be used for meaningful analyses. They are also useful for comparisons with previous modelling studies assuming similar pathways. In general, the rates we determine for the HH model are similar to rates previously inferred for experimental CHO cell data fitted to a HH-like assembly pathway [4], with the exception of addition of light chains to the heavy chain dimer, for which our models predict faster reactions than the CHO model (Table 2). In our model such high rates are required to take account of the fact that the HH and HHL intermediates could not be observed experimentally. At the fitted rates, the assembly reaction can proceed via this pathway with intermediate levels below their experimental detection limit of ∼10^5^ molecules per cell (derived from the experimentally determined detection limit for full antibodies). Thus, we conclude that our observed steady state values are consistent with IgG4 assembly *via* either of the two suggested assembly routes, or with a mixed assembly involving the two routes combined in any proportion.

**Table 2:** Summary of apparent rate constants.

		2N2	2P	4O	4R	Reported range in CHO cells [4]	
**All models**							
_µ_	Growth rate	0.020	0.025	0.036	0.025	0.025–0.035	
K_1_	H.RNA transcription	15.6	107	5	9	38–670	molecules* hour^−1^
K_2_	H.RNA turnover	0	0.4	0.038	0.043	0.09–0.11	hour^−1^
K_3_	L.RNA transcription	1214	5486	46	28	82–1222	molecules* hour^−1^
K_4_	L.RNA turnover	0	0.1	0.026	0.026	0.03–0.06	hour^−1^
**HL model**							
K_5_	H translation	37565	13193	12042	2007	n/a	molecules* RNA^−1^* hour^−1^
K_8_	L translation	1498	169	2140	1129	n/a	molecules* RNA^−1^* hour^−1^
K_7_	H + L −> HL	3.5×10^−6^	9.1×10^−7^	1.5×10^−5^	8.2×10^−8^	n/a	molecule^−1^* hour^−1^
K_10_	L + L −> LL	8.5×10^−6^	1.0×10^−6^	2.5×10^−7^	1.0×10^−7^	n/a	molecule^−1^* hour^−1^
K_16_	HL + HL −> LHHL	8.9×10^−10^	8.9×10^−10^	8.9×10^−10^	8.9×10^−10^	n/a	molecule^−1^* hour^−1^
**HH model**							
K_5_	H translation	37884	7521	8615	827	331–2154	molecules* hour^−1^
K_8_	L translation	1502	170	2213	2141	431–1513	molecules* hour^−1^
K_10_	L + L −> LL	8.5×10^−6^	1×10^−6^	2.5×10^−7^	1×10^−7^	10^−10^–10^−9^	molecule^−1^* hour^−1^
K_19_	H + H −> HH	5.1×10^−7^	1.2×10^−7^	6.1×10^−5^	2.6×10^−8^	10^−7^–10^−1^	molecule^−1^* hour^−1^
K_21_	HH + L −> HHL	6.5	0.49	0.13	0.09	10^−6^–10^−5^	molecule^−1^* hour^−1^
K_22_	HHL + L −> LHHL	6.5	0.49	0.13	0.09	10^−6^–10^−5^	molecule^−1^* hour^−1^

**H  = ** heavy chain; **L = ** light chain.

**Figure 5:**
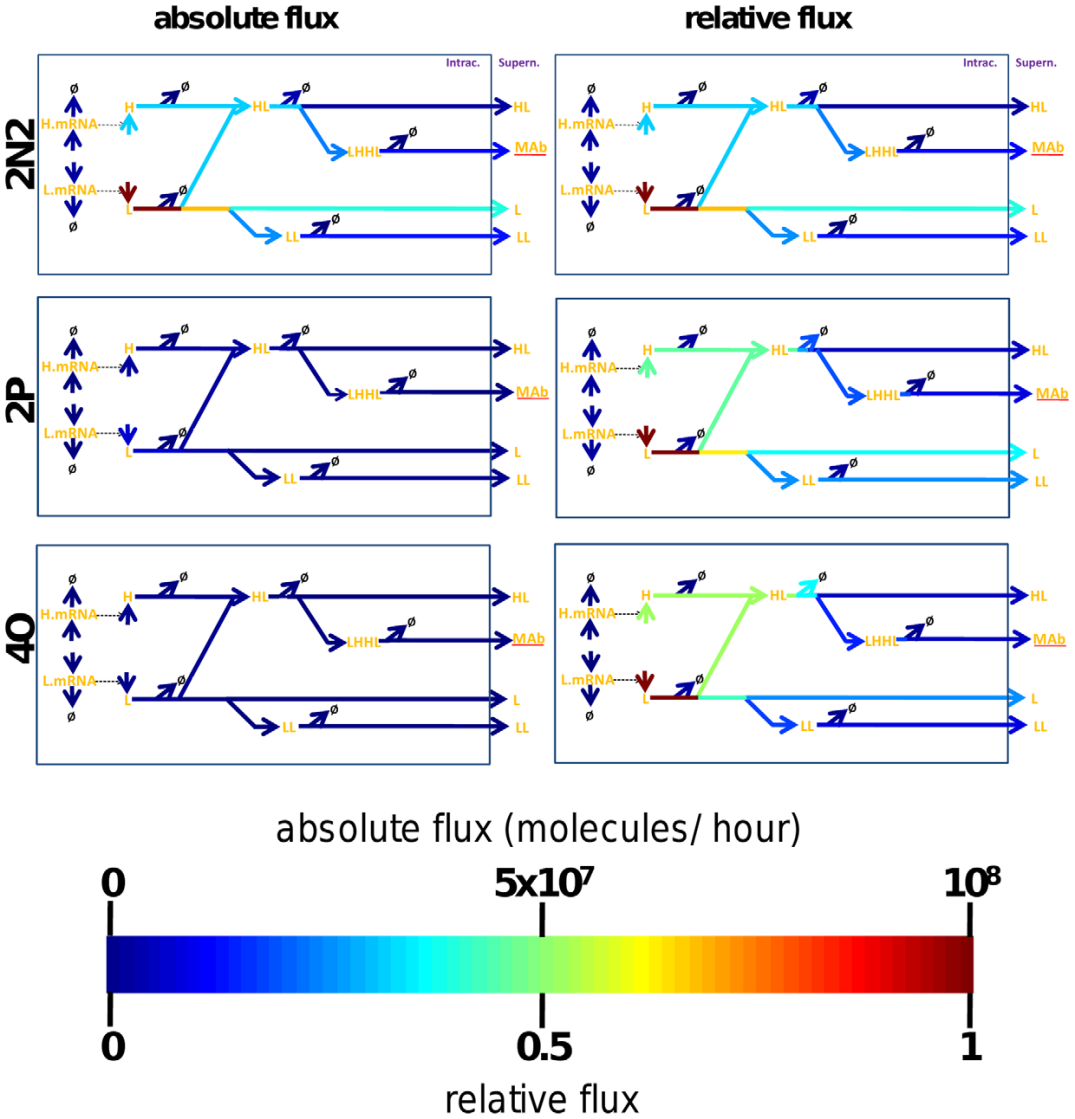
Comparison of pathway fluxes as a function of productivity. The molecular flux through individual reactions was obtained from the biochemical models running at steady state. The colour of the arrows connecting the model species indicates the molecular flux from one species to the next in molecules per time unit. The left hand panel show absolute pathway fluxes for three cell lines. The right hand panel shows relative fluxes, normalised to the highest individual reaction flux observed for each cell line. The diagrams shown are for the “HL” assembly pathway. H.mRNA (heavy chain mRNA), L.mRNA (light chain mRNA), LHHL (assembled antibody), LH (half antibody species), H (heavy chain), HH (heavy chain dimer), HHL (heavy chain dimer + 1 light chain), LL (light chain dimer), L (light Chain), mAb (assembled secreted antibody).

We then used the models to derive the relative flux of material through the different branches of the antibody expression and assembly pathway (shown for the HL model in Fig. 5). Especially when fluxes are normalised to the qmAb of the different cell lines, it becomes apparent that the relative proportion of unproductive export (L, LL and HL species) to productive export (mAb) remains remarkably constant between cell lines of different productivity (shown for the HL pathway in figure 5, but similarly observed with the HH pathway models). This finding would be consistent with all species being exported via a common, non-selective export mechanism, which processes the intracellular species strictly according to their abundance. The ratio of exported species in this scenario is determined by the ratio of free intracellular heavy- and light-chains. This would agree with other studies that have suggested that mAb secretion does not exert a significant limitation upon mAb productivity and that the secretory machinery is not saturated beyond the ER and able to adjust to fluxes in the amount of mAb [4], [46].

**Figure 6:**
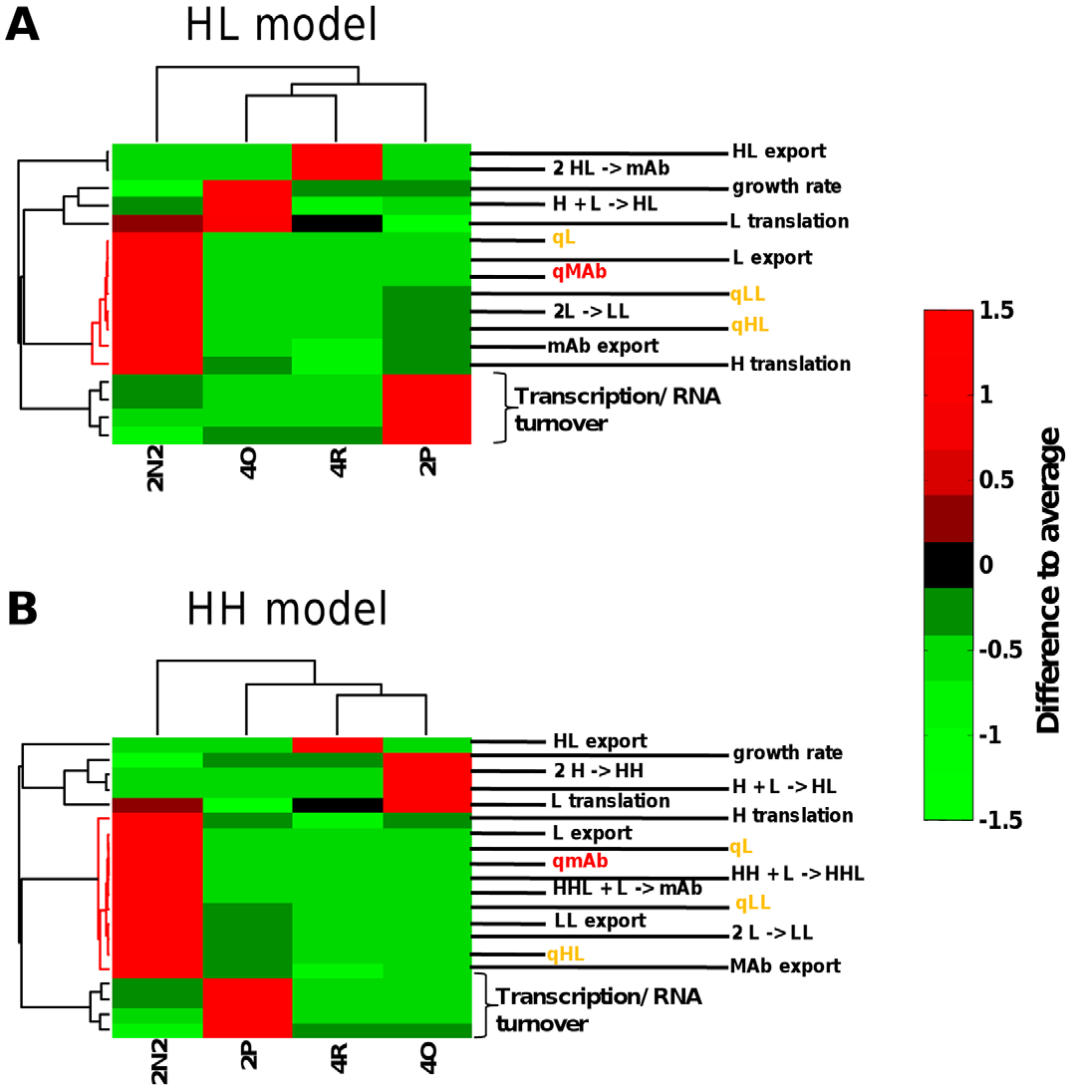
Clustering analysis of cell-line specific parameters. Parameters were obtained from the biochemical models at steady state for each of the four cell lines analysed. Parameters that occur in similar patterns across the different cell lines are closely clustered in this analysis. Statistically significant clusters containing the qmAb parameter are indicated in red. H.mRNA (heavy chain mRNA), L.mRNA (light chain mRNA), LHHL (assembled antibody), LH (half antibody species), H (heavy chain), HH (heavy chain dimer), HHL (heavy chain dimer +1 light chain), LL (light chain dimer), L (light Chain), mAb (assembled secreted antibody).

Since the four cell lines studied here differ significantly in the mAb secretion rates (qmAb), we used the models to investigate whether any of the intracellular parameters are closely linked to this primary parameter. We assessed the similarity in the patterns with which all model parameters occur across the different cell lines by means of clustering analyses. Clustering was based on the correlation coefficients between the different parameter sets, meaning that parameters cluster together if they are highly correlated between the different conditions analysed. Results from this analysis for the HL and HH models are shown in figure 6. In both versions of the model, antibody secretion or qmAb (the primary parameter of interest) forms a cluster with all other secretion rates, as well as with parameters from the later stages of the intracellular assembly process. In contrast, parameters from earlier stages of the assembly process do not tend to fall into this cluster. The sole exception to this rule is the H-translation rate, which appears linked to the qmAb parameter in both the HL and HH model versions (Fig. 6). In summary, these findings indicate that a high qmAb is most likely to occur in cell lines where secretion rates for other species, as well as rates in the late assembly stages, are also high.

We observe an interesting difference in clustering between “secretion” and “export” parameters for individual species. The secretion parameters (qmAb, qL etc) are essentially zero-order rate constants that describe the constant export of molecules as a function of the cell type. In contrast, the export parameters are apparent first-order rate constants that describe export as a function of the intracellular abundance of the exported species. The qmAb, mAb export, qL, L export, qLL, LL export and qHL parameters are all located within the same cluster, in other words, they have a tendency to show similar patterns across the different cell lines. In contrast, the “HL export” parameter is not part of this cluster. This indicates that HL export is functionally unlinked from the abundance of intracellular HL, and may instead be linked to other export activities of the cell. This is further evidence that HL may not actually be exported as HL but rather as non-covalently bound intact antibody but which is observed as an HL molecule under SDS-PAGE conditions as described above.

**Figure 7:**
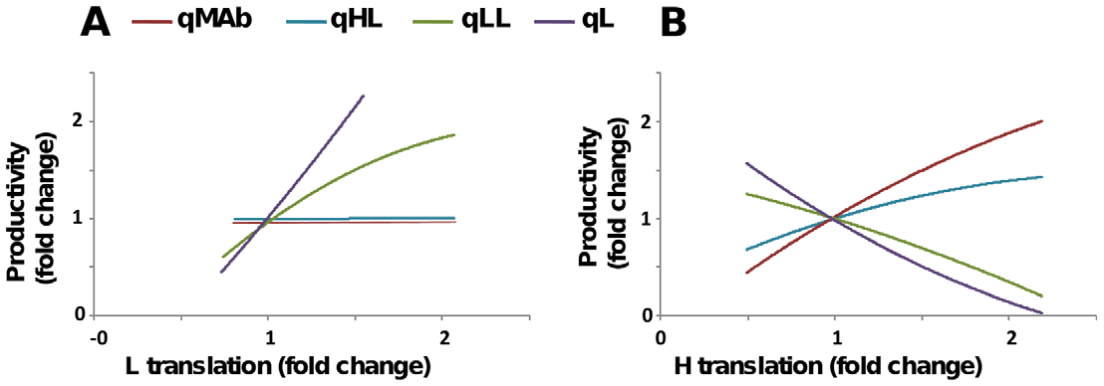
Predicted intervention points for the reduction of by-product export. The predicted effect of variations in increased heavy- (left panel) or light-chain (right panel) expression is illustrated. While variations in light chain expression predominantly affect secretion of the L and LL by-product species, variation in heavy chain expression selectively affects the expression of fully formed antibody. qmAb (assembled antibody secretion rate), qHL (half antibody secretion rate), qLL (light chain dimer secretion rate), qL (light chain secretion rate).

The clustering analysis further indicates that of those intracellular parameters that are amenable to manipulation, heavy-chain translation is the one that is most closely linked to qmAb. In all cell lines investigated total light chain polypeptide produced differs owing to differing transcript levels, however the number of times each individual light chain transcript is translated per hour remains relatively stable between cell lines of varying productivity (Table 2). In contrast, our models show that in addition to differing heavy chain transcript levels the highest producing 2N2 cell line uses each heavy chain transcript for several more rounds of translation per hour than the lower producing cell lines. Thus, if one wished to maximise qmAb and minimise secretion of other species, this would be the most promising parameter to manipulate. In order to explicitly test this prediction in our models we systematically altered heavy-chain translation rates and followed the predicted change in secretion rates for the four secreted species (in our models, an increase in heavy-chain mRNA or in heavy-chain translation has the same effect). For comparison, we performed the same exercise for variations in light-chain production rates. Results from both the biochemical and engineering models predict that increasing light-chain production rates will simply lead to an increase in non-productive qL and qLL, while leaving qHL and qmAb largely unaffected (Fig. 7). In contrast, increasing heavy-chain production is predicted to favourably affect qmAb over the other secretion rates.

The analyses presented so far indicate that both biochemical and engineering-type models are useful to derive information about complex gene expression pathways with limited information on relevant parameters. As a further modelling exercise, we asked whether we could use the engineering model for types of analysis that are classically used in control engineering, but which are not frequently used in the biosciences. A case in point is observability analysis, which effectively investigates if a mapping exists linking the external output(s) of a system directly to all the internal states of the system. This observability testing was conceived by Kalman in the early 1960s [52]. We used this principle to ask whether all thirteen parameters currently under consideration were in-fact required to infer qmAb, or whether a subset of these parameters could be sufficient to predict this parameter. This analysis thus has the potential to simplify and focus future experimental campaigns as well as refining models.

We therefore developed a linearised version of the model, based on linerisation in the region of the steady-state operating point. A series of step response tests were performed which demonstrated good correlation between the behaviour of the linear and nonlinear models. This linear model contained the same number of parameters as the nonlinear counterpart, but had a reduced requirement for parameters that needed to be known to predict qmAb. Observability testing demonstrated that it was possible to determine qmAb from nine rather than the original 13 parameters, all of these being abundance parameters for intracellular species. The engineering analysis thus supports a paradigm that qmAb can be inferred from a reduced set of parameters, which are accessible by sampling at a single time point.

To validate the results from the observability analyses, we tested whether models parameterised with a reduced parameter set could predict qmAb of a cell line not previously used in the construction of the model. We used an NS0 cell line (2X) which had been generated at the same time and from the same parental cell line as the four cell lines used in the construction of the model [35]. Judged by the experimentally determined qmAb, this cell line is a mid producer, in between the 2N2 and 2P cell lines described earlier. When the experimentally determined levels of the intracellular intermediates for this cell line were used to parameterise the derived linear model, a qmAb value was predicted of 3.3×10^5^ molecules per cell per hour, an order of magnitude lower than the experimentally determined value of 0.9×10^6^ molecules per cell per hour (Table 3). In contrast, when the reduced parameter set was used to parameterise the original non-linear model, a qmAb value was predicted of 0.9–1×10^6^ molecules per cell per hour, very close to the experimentally determined value. These results indicate that, while the linearised model was not in itself useful for predicting qmAb, it was useful for exploring dynamic system properties and allowed us to simplify the parameterisation strategy for the full non-linear antibody production model. Overall, this result validates the general modelling strategy, and demonstrates that engineering-type models can be used to predict properties of cell lines beyond those used to generate the training data for model construction.

**Table 3:** Summary of observed parameters for the additional naïve 2X cell line.

		2X	
Steady state levels	H.RNA	n.m.	molecules*cell^−1^
	L.RNA	n.m.	
	H	4.2×10^6^	
	L	2.0×10^6^	
	LL	<0.1×10^6^	
	HL	7.3×10^6^	
	HH	<0.1×10^6^	
	LHH	<0.1×10^6^	
	LHHL	3.7×10^6^	
secretion rates	qL	2.2×10^6^	molecules *cell^−1^*h^−1^
	qLL	3.2×10^6^	
	qmAb	0.9×10^6^	
	qHL	0.9×10^6^	
			
rate constants	Growth	0.019	h^−1^

## Discussion

The detailed characterisation of recombinant protein expression systems is essential for understanding differences between expression cell lines and for the rational design of consistent and stable high producers. While this characterisation is relatively straightforward for single-protein products [40], it is complicated for products with more complex assembly pathways such as monoclonal antibodies. Indeed, due to our limited understanding of mAb antibody assembly in industrially relevant cells for different IgG isotopes, it is currently not possible to fully define the limitations upon mAb production and develop cell engineering strategies based upon the analysis of the gene expression pathway to enhance the ability of these systems to synthesise, assemble and secrete antibodies. In this study, we have developed and investigated modelling approaches to describe the gene expression pathway of a model mAb and as a tool to define cell engineering strategies to improve qmAb or as a means of identifying observers of high producing cell lines.

Modelling approaches have been used in the past for the characterisation of antibody assembly pathways [4], [38], [43]. These studies made predictions with respect to the most likely assembly routes and points of control within the system. An emerging theme from these studies is that limitations are exerted in a heterogeneous manner depending on the cell line in question. For example, O'Callaghan *et al*. found that mRNA transcription, mRNA stability, folding of assembly intermediates, and secretion rates could all fully or partially limit qmAb in CHO isolates exhibiting different productivity [4]. Our studies agree with these previous analyses, showing cell line specific limitations whilst also highlighting specific parameters that could be used as observers to predict and screen for cell lines capable of high cell specific productivity. Indeed, given such heterogeneity of control, the rational design of consistently super-expressing cell lines is clearly a difficult problem and as suggested by O'Callaghan and colleagues likely to be cell line specific [4].

In order to model the IgG4 synthesis and assembly pathway we had to consider two potential assembly routes for IgGs. There have been no definitive experimental reports describing the assembly of IgG4 molecules. Although the HH model had previously been suggested as the most likely assembly route [4], our experimental data did not allow us to unambiguously identify the assembly pathway in our system. Indeed, our observed steady state values were consistent with IgG4 assembly *via* either of the two suggested assembly routes, or with a mixed assembly involving the two routes combined in any proportion, as both models could be tuned to reproduce our experimentally observed steady-state data. This suggests that the assembly process itself, and the different chaperones and folding requirements for each of these pathways, are unlikely to be the major limiting determinants in qmAb production.

Recent studies have shown that manipulation of the secretion machinery within recombinant cell lines can increase cell specific secretion rates [26], [53], [54], [55]. O'Callaghan *et al*. reported that the rate of mAb secretion varied widely across a panel of CHO cell lines, and although this did not correlate with qmAb, they suggested secretory engineering as a possible cell line specific engineering approach [4]. Our modelling data are consistent with the fully assembled IgG4 molecule (LHHL) and the off target species (L, LL, HL) all being exported via a common, non-selective export mechanism, which processes the intracellular species strictly according to their abundance. As a result, our data and models predict that any strategies to improve secretion would result in the increased secretion of off target species in addition to the fully assembled molecule, and that the ratio of exported species would remain the same.

The ratio of active product to inactive by-products is an important expression system parameter as it determines the downstream processing behaviour of antibody-based therapeutics. An alternative strategy suggested by our modelling studies is to manipulate points earlier in the pathway by altering the ratio of free intracellular heavy- and light-polypeptide chains. Model derived translation rates identified that the highest producing cell line utilises each heavy chain transcript several fold more times per hour than in the lower producing cell lines and *in-silico* experiments using our model predict increased productivity in all cell lines by increasing heavy chain translation rates. The differential usage of heavy chain transcripts by the highest producing cell line suggests possible cell specific optimisation of the complex cellular translation machinery. In contrast to manipulating secretory activity directly, optimisation of the ratio of free intracellular heavy- and light-chains is predicted to increase qmAb specifically whilst reducing the secretion of the undesirable off target products (qLL and qL, Fig. 7).

Finally, we applied engineering principles to our models in the form of observability analysis in order to indentify a minimum set of parameters required to define cell lines with high qmAb. Our analyses suggest that qmAb may be fully predictable from measurements of a reduced set of solely intracellular species, and we demonstrated the validity of this approach experimentally. In conclusion, modelling approaches can be used to both identify high-producing cell lines and to manipulate the gene expression machinery with the aim of further improving productivity.

## Methods

### Experimental

#### Cell lines and cell culture

The four mouse myeloma (NS0) cell lines used in this study, which are stably expressing IgG_4_ mAb cB72.3 with glutamine synthetase as a selectable marker, have been described previously 35]. The cell lines were generously provided by Lonza Biologics plc (Slough, UK) and were routinely cultured as previously described 19]. For time course studies n = 9 E250 flasks (n = 3 control, n = 3 cycloheximide treatment and n = 3 actinomycin D treatment flasks) were seeded at 0.2×10^6^ cells mL^−1^ in a 50 ml culture volume and maintained as suspension cultures at 36.5°C, 5% CO_2_ and 100 rpm. Cell samples were taken daily for supernatant analysis to determine qMab values and cell counts performed using a Vi-CELL 1.01 (Beckman Coulter, High Wycombe, UK). At mid-exponential phase (96 h post-inoculation) cells were harvested and mRNA and protein analysis undertaken for final model parameterisation as described below.

#### *In Vitro* Transcription of HC and LC mRNA

For *in vitro* transcription of HC and LC mRNA, primers were designed to either side of the region of interest with a T7 promoter sequence added on the forward primer. Amplification of HC and LC fragments was performed by standard PCR using High Fidelity *Taq* polymerase (Roche, Burgess Hill, UK) with the following amplification protocol; 95°C for 5 min, followed by 35 cycles of 95°C for 30 s, 55°C for 30 s and 72°C for 1 min, with a final extension at 72°C for 10 min. PCR products were subsequently gel purified and used as a template in T7 RiboMax Express large scale RNA production system (Promega, Southampton, UK). Following transcription, template DNA was removed by DNase1 digestion and the RNA product purified using an RNeasy mini kit (Qiagen Ltd). Integrity of the transcription product was verified on denaturing polyacrylamide gels.

#### RNA Extraction and Quantitative Real Time PCR

Total RNA was extracted from NS0 cell lines using the RNeasy extraction kit (Qiagen Ltd, UK) with on-column DNAse treatment according to the manufacturer's instructions. The expression level of HC and LC mRNA transcripts was determined by use of gene specific primers and quantitative real time reverse transcription-PCR (qRT-PCR) as previously described 40]. mRNA quantification was carried out using mRNA from n = 3 control samples per cell line. A standard curve was prepared in duplicate from *in vitro* transcribed RNAs in order to calculate actual mRNA levels. Samples were normalised to the housekeeping gene β-actin and mRNA copy numbers extrapolated from the standard curve. For mRNA half-life measurements the transcription inhibitor actinomycin D (final concentration 4 mM) was added at T0 to triplicate biological cultures and relative mRNA levels subsequently determined after 0, 0.5, 1.2, 2, 2.4, 4 and 6 h and mRNA half-lives determined as previously described 40].

#### Cell Extracts and Western Blotting

Intracellular proteins were prepared in RIPA buffer as described (Harlow and Lane 1988), sonicated (16 µm peak-to-peak, 3×5 s) and supplemented with 5x gel loading buffer [56]. Culture supernatants were cleared by centrifugation at 13000 x rpm for 10 min at 4°C and supplemented with 5x gel loading buffer. Samples were resolved by SDS-PAGE on a 10% non-reducing gel and western blotting was performed using a standard semi-dry method [57]. Detection of both intracellular and supernatant species were performed using a rabbit anti-human IgG whole molecule (1∶1000), rabbit anti-human IgG γ chain (1∶500) from Sigma-Aldrich or goat anti-human kappa light chain (1∶1000) from Bethyl Laboratories (Montgomery, USA) as indicated within the manuscript. Primary antibodies were detected using a horse radish peroxidise (HRP) conjugated goat anti-rabbit IgG antibody (Sigma-Aldrich) or rabbit anti-goat HRP (Santa Cruz Biotechnology, USA) as appropriate and visualised using ECL reagent (GE Healthcare Life Sciences, Little Chalfont, UK). Western blot images were analysed with the AIDA image analyser software (Raytest Ltd, UK) and quantification of absolute levels performed as described [49] using purified IgG_4_ as a standard.

#### mAb Species Secretion Rate

Cell specific productivity (q_Mab_) was calculated using absolute quantification of each mAb species by western blotting across day 0 to day 4 of culture and the time integral of viable cells (IVC) [58], [59]. Calculations used were.

Average viable cell concentration (*VCC*)
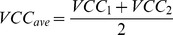



Change in *VCC_ave_* between *t_1_* and *t_2_*

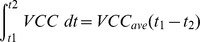



Time integral of viable cells (*IVC*)
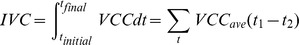



Cell specific productivity
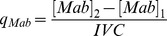



Stability of the intact IgG_4_ species during gel electrophoresis was assessed by addition of the alkylating agent N-ethylmaleimide (NEM) (20 mM final concentration) or the sulfhydryl-reactive homobifunctional crosslinker 1,4-bismaleimidyl-2,3-dihydroxybutane (BMDB, Pierce Biosciences) (0.2 mM final concentration) to both the supernatant and the RIPA buffer immediately after sample collection. Samples were subsequently analysed by standard western blotting.

#### mAb Species Stability

Stability of the ratio of intact IgG_4_ species to half mAb in culture supernatant was tested by incubating cleared supernatant in a 37°C sterile environment for up to 96 h post collection, resolving by SDS-PAGE and detection by Coomassie blue staining.

### SimBiology model

#### Implementation

The differential equation systems provided in File S3 were used to construct a standard biochemical model in the Matlab Simbiology toolbox (version 2009a, MathWorks, Cambridge, UK). Details of the model are shown in figure 4A. The model was parameterised by calculating apparent first order rate constants from the observed productivity data and levels of the intracellular species, under the assumption of protein turnover for these species being negligible compared to the dilution through cell growth as described in the results section. Models were run using the standard ode45 (Dormand-Prince) solver with typical simulation times of 25,000 seconds.

#### Data Clustering

Apparent first-order rate constants for all chemical transitions in the system and for each cell-line were hierarchically clustered using the Matlab *clustergram* procedure. Clustering was performed based on Spearman's rank correlation relating the parameter sets for each cell line.

### Simulink model

#### Implementation

The engineering model was developed in Simulink and included all possible interconnections between the parameters including nonlinear interconnections. Where available, rate constants were used as determined experimentally. Any unknown rate constants were determined via simple least squares fit at steady-state conditions. This latter procedure was carried out independently for the HH, HL and combined model to provide information on the likely variation in rate constant to benchmark the models for robustness. It was possible to determine rate constants consistent across the three models. Having developed a nonlinear model, the model was then linearised in the region of the steady state operating point. A series of step response tests were performed which demonstrated good correlation between the behaviour of the linear and nonlinear models. This linear model contained the same number of parameters as the nonlinear counterpart.

#### Observability analysis

Having obtained and validated the linear model representation, further analysis was undertaken to infer to what degree a model of lower order could be used to infer qmAb. The observability testing demonstrated that it is possible to construct qmAb from nine, rather than the 13 parameters present in the original model. Step response tests on this reduced order model correlated well with the step response tests performed on both the nonlinear and linear models in the region of steady state operation.

#### Model Validation

We validated our models with a naive cell line to determine whether the models could predict the qmAb from measurement of the parameters suggested by the minimal observability analysis. We initially estimated K8 using the minimal parameters measured for the naïve cell line for the HH and HL models and then for the combined model used a coarse average of the two estimates. As measurements were not experimentally taken to enable k1 and k3 to be determined directly, we used the following equations to determine suitable values from the measured data;
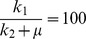
and



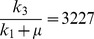



The linear model was then generated as before, with the above assumptions, the new measured data and the previous model. The observability analysis was carried out on the linear model exactly as previously described with the intent of identifying the species that determine the final output. Again the number of observable states was found to be nine from the naive cell line experimental results.

## Supporting Information

File S1 **Growth profiles of all 4 cell lines throughout batch culture and evidence in 1 L spinner culture of consistent antibody production at the sampling time point.**
(PDF)

File S2 **Western blot data of n = 3 2N2 samples using anti-heavy chain, anti-whole chain and anti-light chain antibodies on either reducing or non-reducing SDS-PAGE gels.** Example of a western blot standard curve and quantification is additionally provided.
(PDF)

File S3 **Details of the reaction schemes and differential equation systems used for establishing computational models.**
(PDF)

File S4 **Matlab source code files (M-files).**
(RAR)

File S5 **Raw data used for model construction and validation.**
(XLS)
