# Supplementary material for: Experimental and In Silico Modelling Analyses of the Gene Expression Pathway for Recombinant Antibody and By-Product Production in NS0 Cell Lines
Source: PLoS One. 2012 Oct 10;7(10):e47422. doi: 10.1371/journal.pone.0047422 (PMC3468484; doi:10.1371/journal.pone.0047422)
Supplement: File S1 — Growth profiles of all 4 cell lines throughout batch culture and evidence in 1 L spinner culture of consistent antibody production at the sampling time point. (PDF) [file pone.0047422.s001.pdf]

Mead *et al.*

Supplementary file 1

Growth profiles of all 4 cell lines throughout batch culture and evidence in 1L spinner culture for consistent antibody production at the sampling time point.

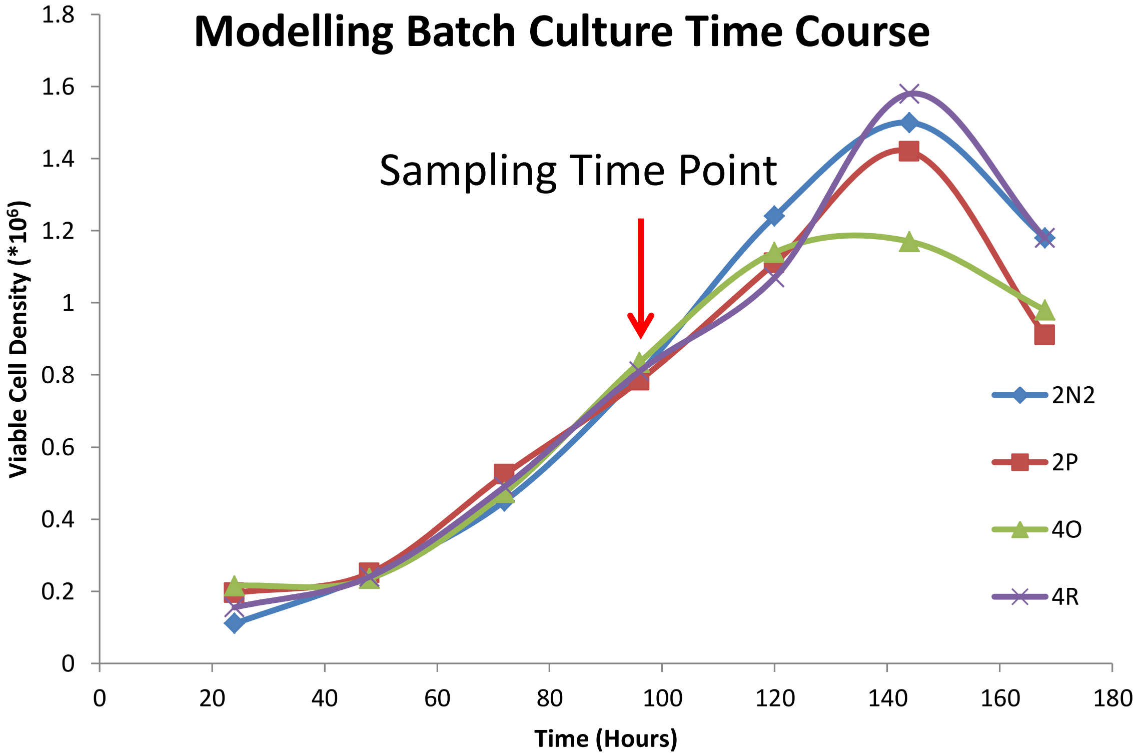

Supplementary File 1, Figure 1. Full growth curves for all four cell lines used in this study in E250 shake flasks. Sampling time point was during mid-exponential phase for all 4 cell lines.

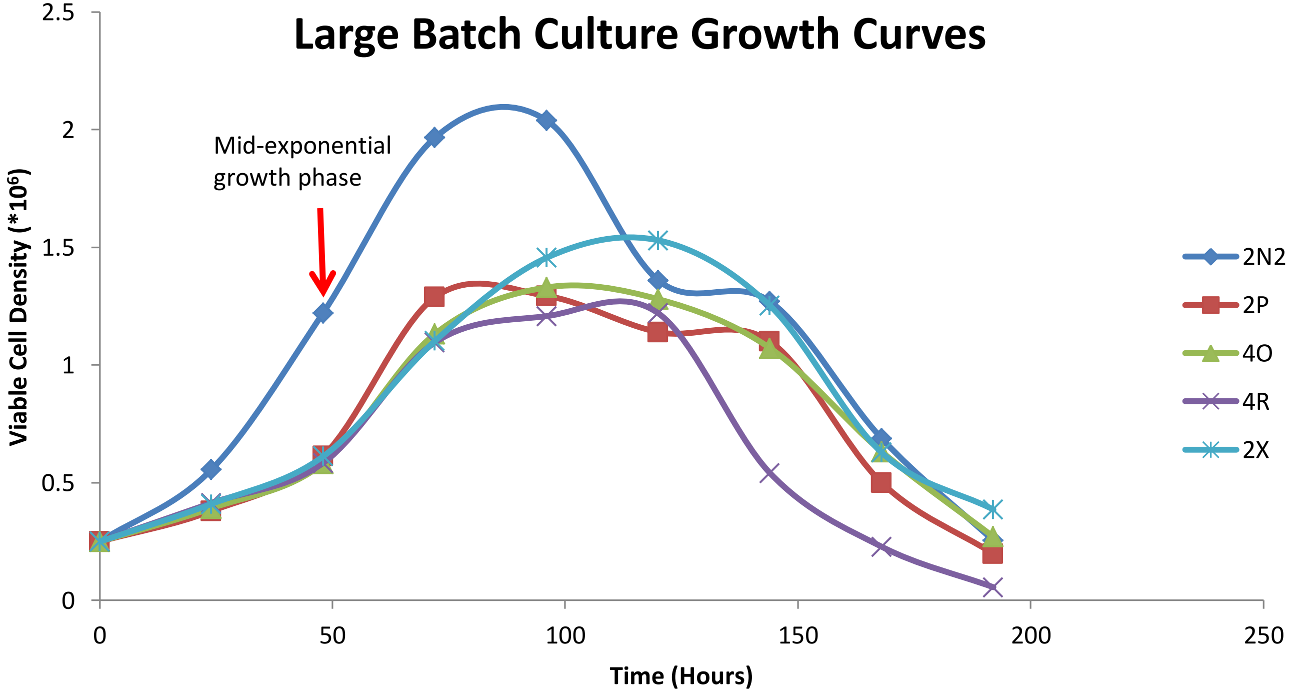

Supplementary File 1, Figure 2. Full growth curves in 1L spinner culture for all five cell lines (including 2x used for model validation) from a previous study (ref 35). Due to slightly different growth conditions and seeding densities mid-exponential growth is at approximately 48 hours for these samples.

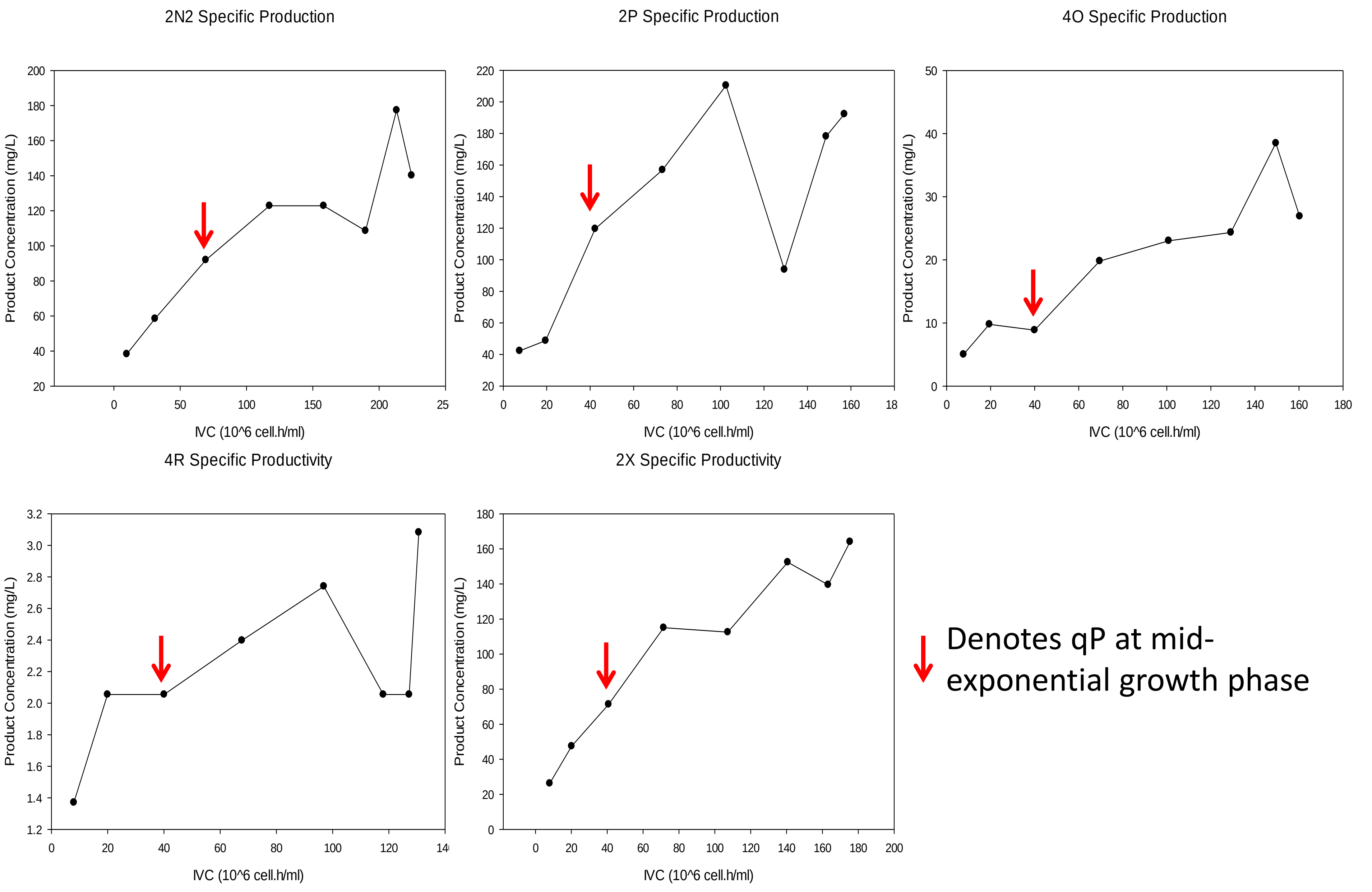

Supplementary File 1, Figure 3. Specific productivity curves for all 5 cell lines.
