# Supplementary material for: Experimental and In Silico Modelling Analyses of the Gene Expression Pathway for Recombinant Antibody and By-Product Production in NS0 Cell Lines
Source: PLoS One. 2012 Oct 10;7(10):e47422. doi: 10.1371/journal.pone.0047422 (PMC3468484; doi:10.1371/journal.pone.0047422)
Supplement: File S2 — Western blot data of n = 3 2N2 samples using anti-heavy chain, anti-whole chain and anti-light chain antibodies on either reducing or non-reducing SDS-PAGE gels. Example of a western blot standard curve and quantification is additionally provided. (PDF) [file pone.0047422.s002.pdf]

Mead *et al.*

Supplementary file 2

Western blot data against n=3 2N2 samples using anti-heavy chain, anti-whole chain and anti-light chain antibodies on either reducing or non-reducing SDS-PAGE gels. Example of a Western blot standard curve and quantification is additionally provided.

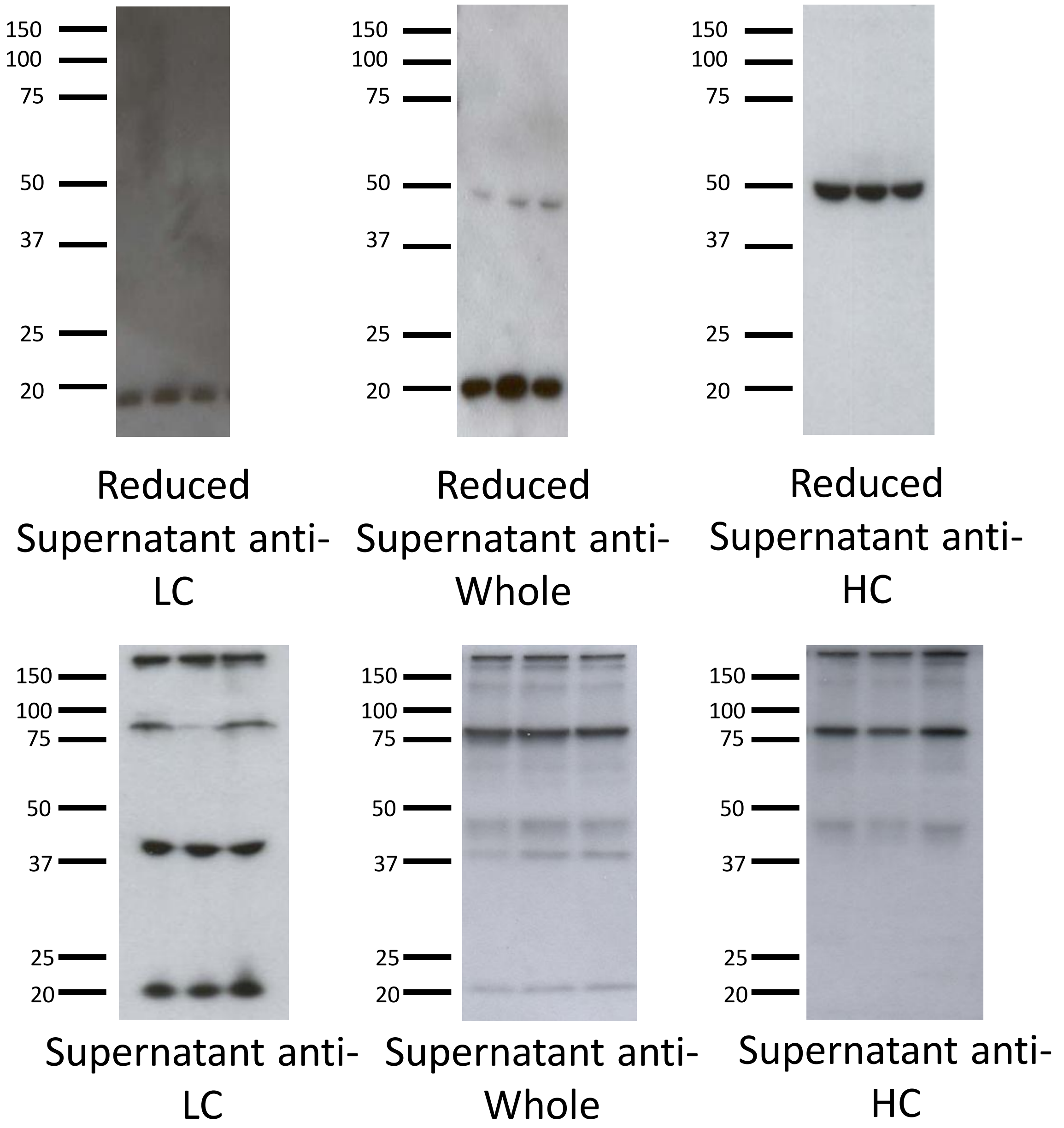

Supplementary File 2, Figure 1. Western blot using anti-LC, anti-whole chain and anti-HC antibodies on reduced and non-reduced gels.

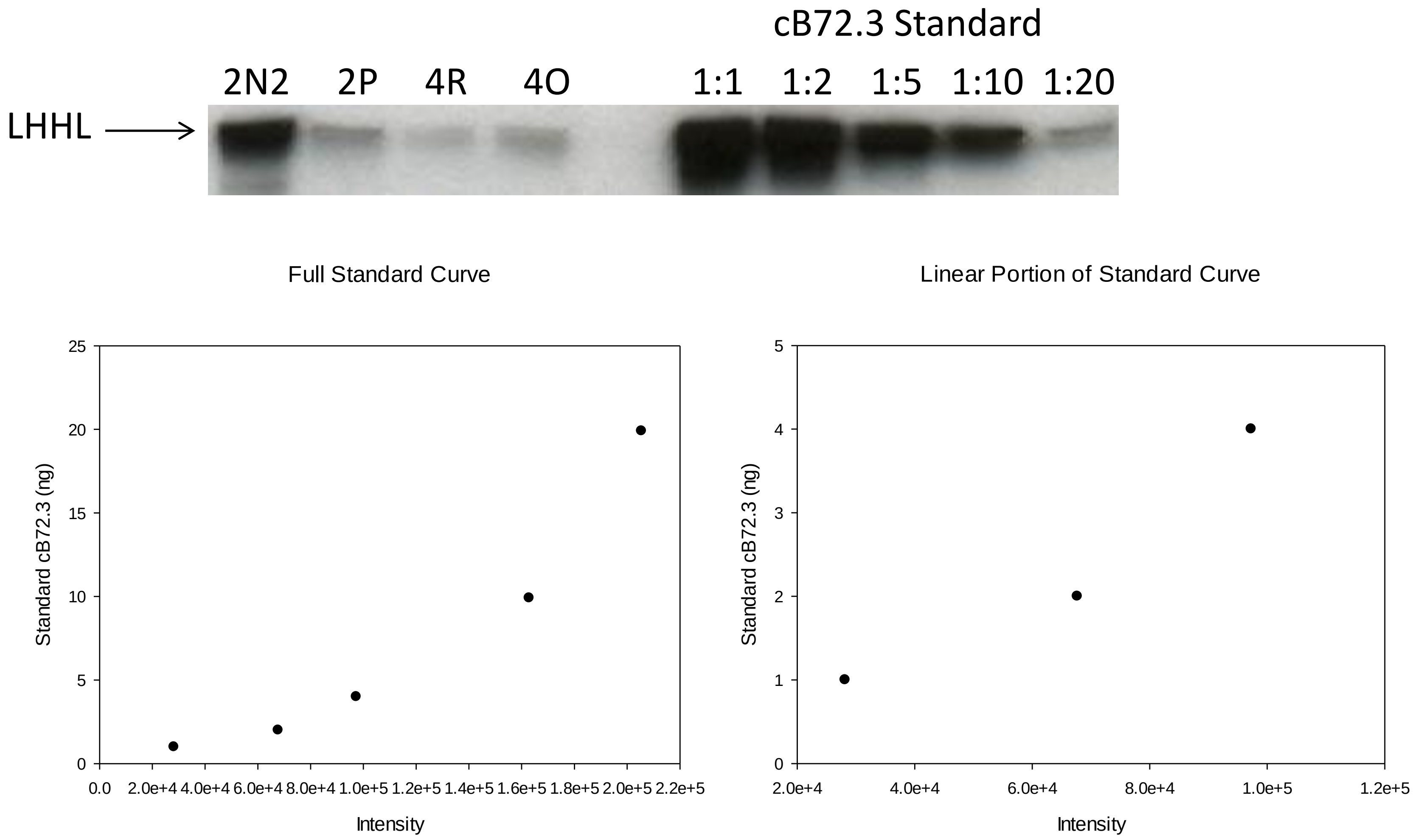

Supplementary File 2, Figure 2. Representative western blot quantification of intact antibody using cB72.3 standard. Only values falling in the linear range of the standards were used. Several different exposure times were performed alongside different standard ranges in order to obtain accurate quantification for each species within each cell line.
