# Supplementary material for: Experimental and In Silico Modelling Analyses of the Gene Expression Pathway for Recombinant Antibody and By-Product Production in NS0 Cell Lines
Source: PLoS One. 2012 Oct 10;7(10):e47422. doi: 10.1371/journal.pone.0047422 (PMC3468484; doi:10.1371/journal.pone.0047422)
Supplement: File S3 — Details of the reaction schemes and differential equation systems used for establishing computational models. (PDF) [file pone.0047422.s003.pdf]

Mead *et al.*

Supplementary file 3

Pathway diagrams and differential equation systems for the different antibody assembly models considered in this study.
In pathway diagrams, productive reactions (leading to secreted full antibody) are shown in red.

HH model

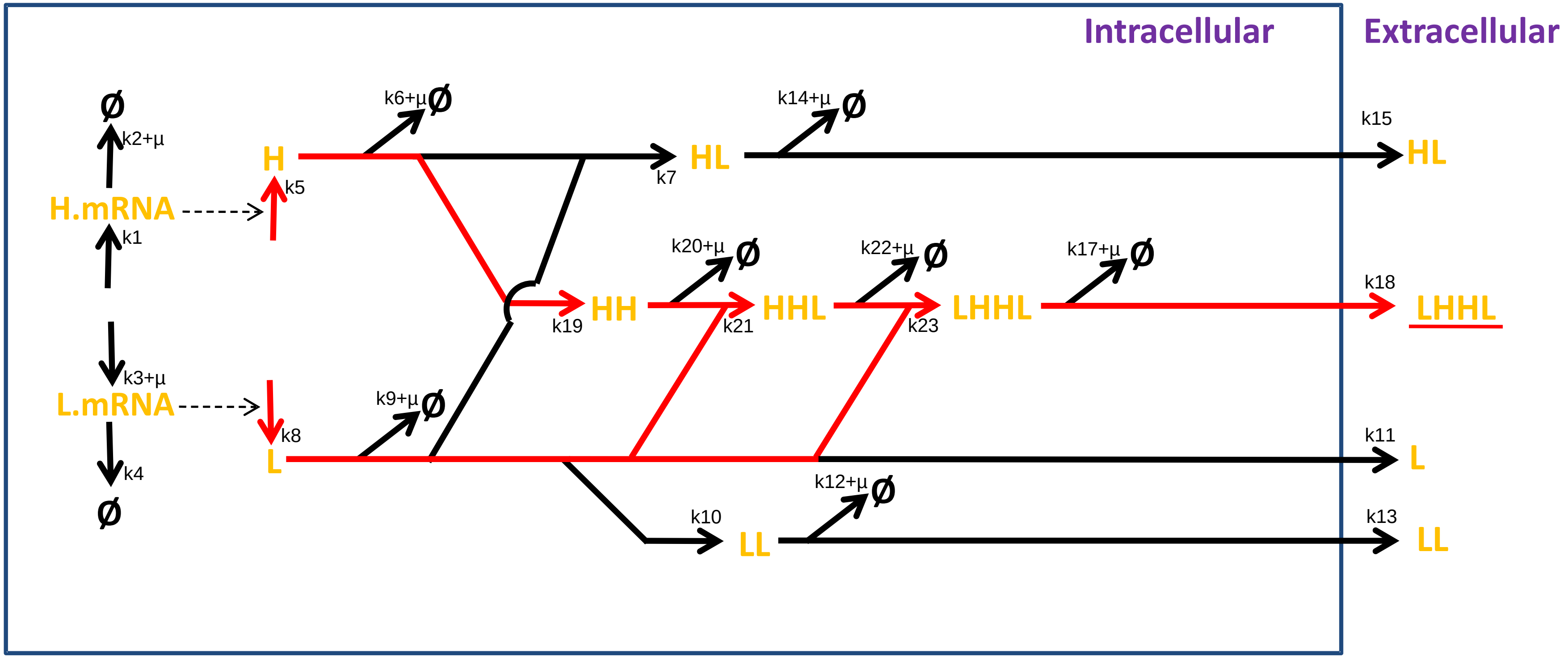

Species	Name	Eq.
S1	H.RNA	$dS1/dt = k1 - (k2 + \mu)*S1$
S2	L.RNA	$dS2/dt = k3 - (k4 + \mu)*s2$
S3	H	$dS3/dt = k5*S1 - (k6 + \mu)*S3 - k19*S3*S3 - k7*S3*S4$
S4	L	$dS4/dt = k8*S2 - (k9+\mu)*S4 - k10*S4*S4 - k11*S4 - k21*S10*S4 - k23*S11*S4 - k7*S3*S4$
S5	L(Medium)	$dS5/dt = k11*S4$
S6	LL	$dS6/dt = k10*S4*S4 - (k12 + \mu)*S6 - k13*S6$
S7	LL(Medium)	$dS7/dt = k13*S6$
S8	HL	$dS8/dt = k7*S3*S4 - (k14 + \mu)*S8-k15*S8$
S9	HL(Medium)	$dS9/dt = k15 * S8$
S10	HH	$dS10/dt = k19*S3*S3 - (K20 + \mu)*S10 - k21*S10*S4$
S11	LHH	$dS11/dt = K21*S10*S4 - (k22 + \mu)*S11 - k23*S11*S4$
S12	LHHL	$dS12/dt = k23*S11*S4 - (k17 + \mu)*S12 - k18*S12$
S13	LHHL(Medium)	$dS13/dt = k18*S12$

HL model

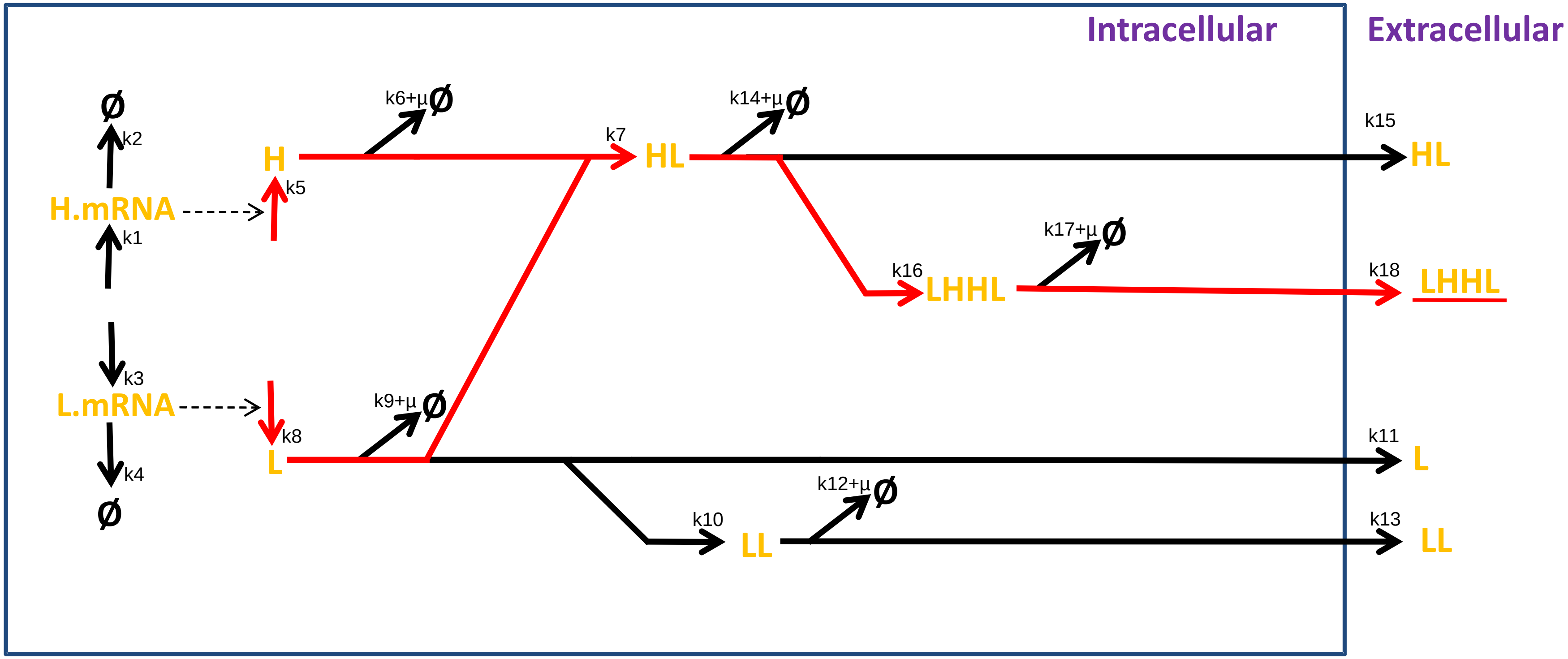

Species	Name	Eq.
S1	H.RNA	dS1/dt = k1 – (k2 + μ)*S1
S2	L.RNA	dS2/dt = k3 – (k4 + μ)*S2
S3	H	dS3/dt = k5*S1 – (k6+μ)*S3 – k7*S3*S4
S4	L	dS4/dt = k8*S2 – (k9+μ)*S4 – k7*S3*S4 – k10*S4*S4 – k11*S4
S5	L(Medium)	dS5/dt = k11*S4
S6	LL	dS6/dt = k10*S4*S4 – (k12+μ)*S6 - k13*S6
S7	LL(Medium)	dS7/dt = k13*S6
S8	HL	dS8/dt = k7*S3*S4 – (k14+μ)*S8 – k15*S8 – k16*S8*S8
S9	HL(Medium)	dS9/dt = k15*S8
S12	LHHL	dS12/dt = k16*S8*S8 – (k17+μ)*S12 – k18*S12
S13	LHHL(Medium)	dS13/dt = k18*S12

combined model

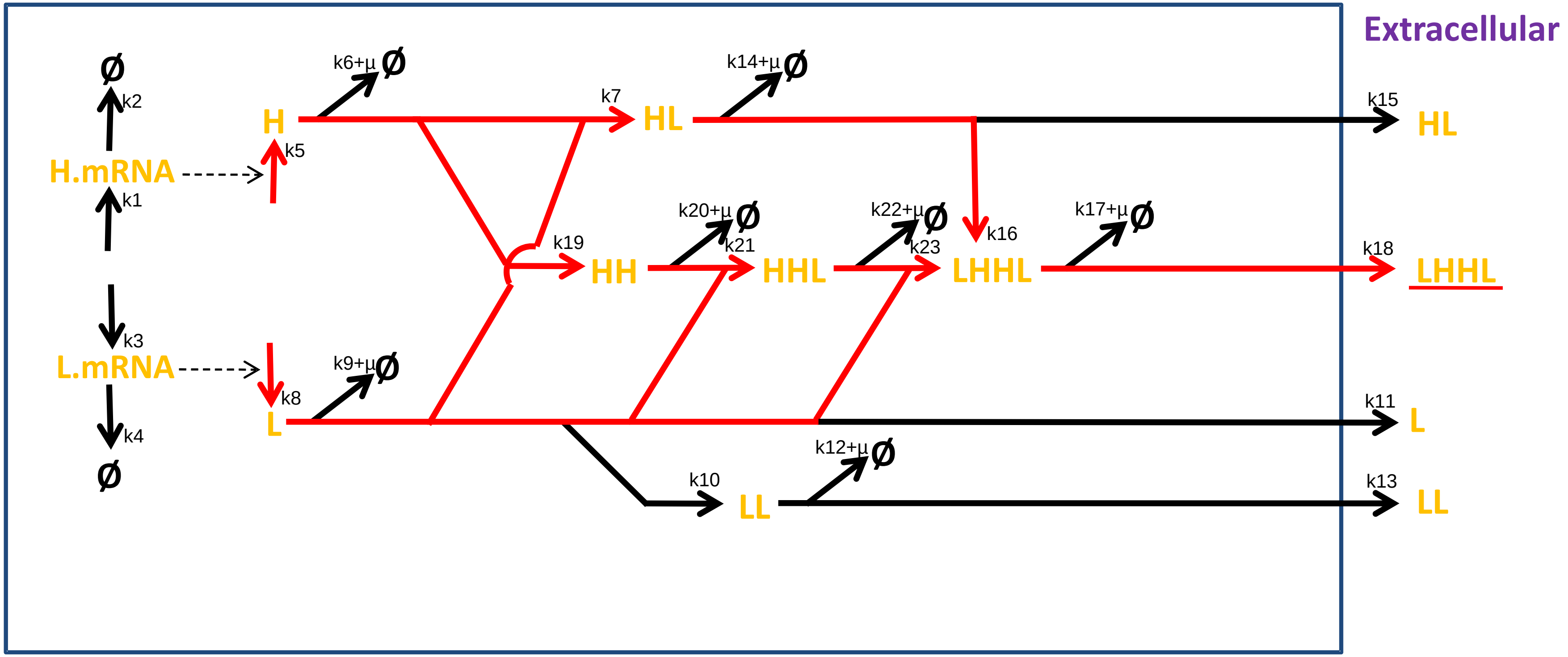

Species	Name	Eq.
S1	H.RNA	$dS1/dt = k1 - (k2+\mu)*S1$
S2	L.RNA	$dS2/dt = k3 - (k4+\mu)*s2$
S3	H	$dS3/dt = k5*S1 - (k6+\mu)*S3 - k7*S3*S4 - k19*S3*S3$
S4	L	$dS4/dt = k8*S2 - (k9+\mu)*S4 - k7*S3*S4 - k10*S4*S4 - k11*S4 - k21*S10*S4 - k23*S11*S4$
S5	L(Medium)	$dS5/dt = k11*S4$
S6	LL	$dS6/dt = k10*S4*S4 - (k12+\mu)*S6 - k13*S6$
S7	LL(Medium)	$dS7/dt = k13*S6$
S8	HL	$dS8/dt = k7*S3*S4 - (k14+\mu)*S8 - k16*S8*S8 - k15*S8$
S9	HL(Medium)	$dS9/dt = k15 * S8$
S10	HH	$dS10/dt = k19*S3*S3 - (k20+\mu)*S10 - k21*S10*S4$
S11	LHH	$dS11/dt = k21*S10*S4 - (k22+\mu)*S11 - k23*S11*S4$
S12	LHHL	$dS12/dt = k23*S11*S4 + k16*S8*S8 - (k17+\mu)*S12 - k18*S12$
S13	LHHL(Medium)	$dS13/dt = k18*S12$

James model

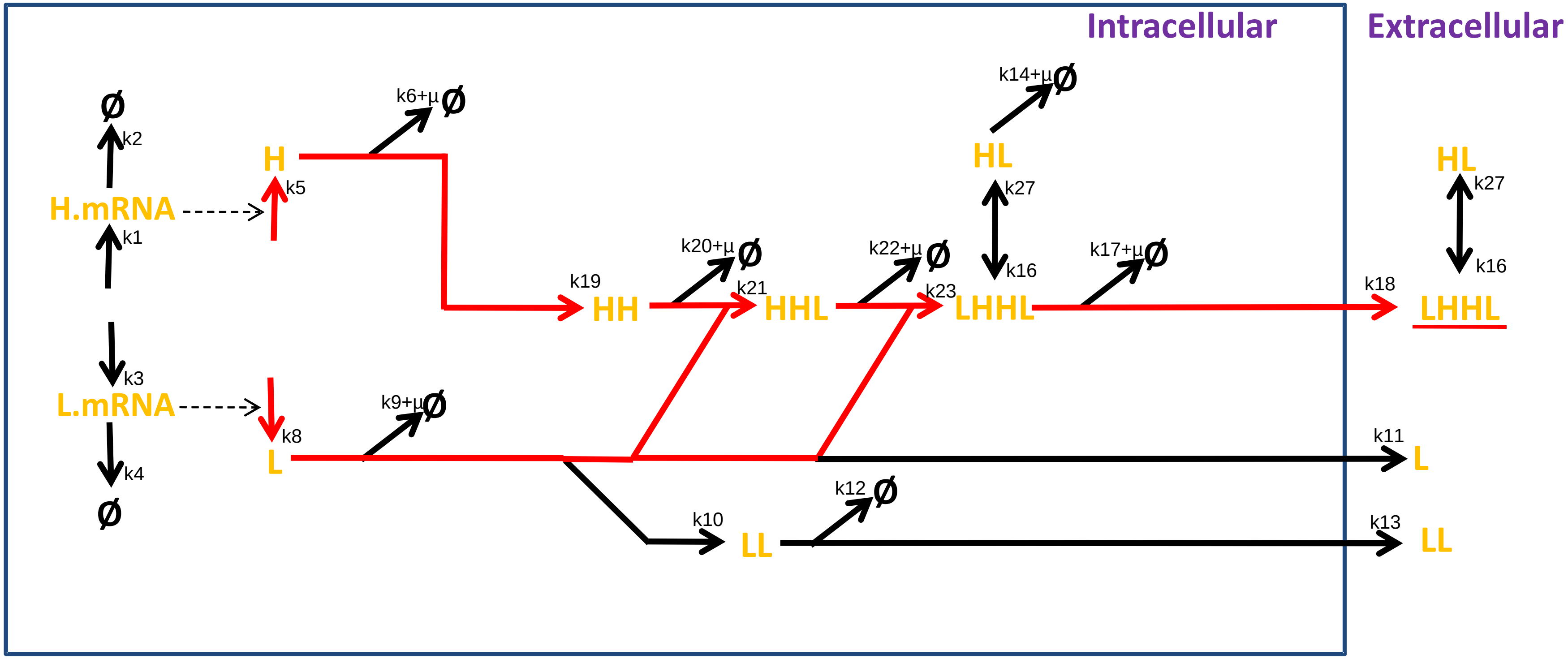

Species	Name	Eq.
S1	H.RNA	dS1/dt = k1 – (k2+μ)*S1
S2	L.RNA	dS2/dt = k3 – (k4+μ)*S2
S3	H	dS3/dt = k5*S1 – (k6+μ)*S3 – k19*S3*S3
S4	L	dS4/dt = k8*S2 – (k9+μ)*S4 – k10*S4*S4 – k11*S4 – k21*S10*S4 – k23*S11*S4
S5	L(Medium)	dS5/dt = k11*S4
S6	LL	dS6/dt = k10*S4*S4 – (k12+μ)*S6 – k13*S6
S7	LL(Medium)	dS7/dt = k13*S6
S8	HL	dS8/dt = k27*S12 – (k14+μ)*S8 – k16*S8*S8
S9	HL(Medium)	dS9/dt = k27*S13 – k16*S9*S9
S10	HH	dS10/dt = k19*S3*S3 – (k20+μ)*S10 – k21*S10*S4
S11	LHH	dS11/dt = K21*S10*S4 – (k22+μ)*S11 – k23*S11*S4
S12	LHHL	dS12/dt = k23*S11*S4 + k16*S9*S9 – (k17+μ)*S12 – k18*S12 – k27*S12
S13	LHHL(Medium)	dS13/dt = k18*S12 – k27*S13

Nomenclature

Rate constants:

	type	description	unit
μ	rate constant	cell growth rate	h^{-1}
k1	rate	H.RNA transcription	mRNAs h^{-1}
k2	rate constant	H.RNA turnover	h^{-1}
k3	rate	L.RNA transcription	mRNAs h^{-1}
k4	rate constant	L.RNA turnover	h^{-1}
k5	rate constant	H translation	molecules $mRNA^{-1}$ h^{-1}
k6	rate constant	H turnover	h^{-1}
k7	rate constant	H + L ->HL	$molecule^{-1}$ h^{-1}
k8	rate constant	L translation	molecules $mRNA^{-1}$ h^{-1}
k9	rate constant	L turnover	h^{-1}
k10	rate constant	L + L -> LL	$molecule^{-1}$ h^{-1}
k11	rate constant	L export	h^{-1}
k12	rate constant	LL turnover	h^{-1}
k13	rate constant	LL export	h^{-1}
k14	rate constant	HL turnover	h^{-1}
k15	rate constant	HL export	h^{-1}
k16	rate constant	HL + HL -> LHHL	$molecule^{-1}$ h^{-1}
k17	rate constant	LHHL turnover	h^{-1}
k18	rate constant	LHHL export	h^{-1}
k19	rate constant	H + H -> HH	$molecule^{-1}$ h^{-1}
k20	rate constant	HH turnover	h^{-1}
k21	rate constant	HH + L -> LHH	$molecule^{-1}$ h^{-1}
k22	rate constant	LHH turnover	h^{-1}
k23	rate constant	LHH + L -> LHHL	$molecule^{-1}$ h^{-1}
k27	rate constant	LHHL -> HL+HL	h^{-1}
k28	rate constant	HL + HL -> LHHL	$molecule^{-1}$ h^{-1}

Species

S1	H.mRNA
S2	L.mRNA
S3	H
S4	L
S5	L(Medium)
S6	LL
S7	LL(Medium)
S8	HL
S9	HL(Medium)
S10	HH
S11	LHH
S12	LHHL
S13	LHHL(Medium)
S14	LH:HL
S15	LH:HL(Medium)
